# Talking about links between sexually transmitted infections and infertility with college and university students from SE England, UK: a qualitative study

**DOI:** 10.1186/1742-4755-10-47

**Published:** 2013-09-11

**Authors:** A Lauren R Goundry, Emma R Finlay, Carrie D Llewellyn

**Affiliations:** 1 Department of Primary Care & Public Health, Brighton and Sussex Medical School, Falmer Brighton, Room 317 Mayfield House, East Sussex BN1 9PH, UK

**Keywords:** Sexually transmitted infection, Infertility, Adolescents, Focus group, Qualitative

## Abstract

**Background:**

Sexually transmitted infections (STIs) such as chlamydia and gonorrhoea are largely symptomless diseases which, left untreated, can result in serious complications including infertility. Fertility problems currently affect approximately one in seven couples in the UK and there is increasing demand for couples seeking reproductive technologies. Young people are at greatest risk of contracting STIs, therefore this study aimed to identify young people’s knowledge and beliefs about the link between untreated STIs and infertility.

**Methods:**

Focus groups were conducted with participants aged 16–24 years old inclusive in college or university settings in the SE of England. Groups were quota sampled on the basis of age and gender. A topic guide was used. The data were analysed using a framework analysis approach.

**Results:**

Ten single-sex focus groups were conducted with sixty participants: six groups of college students and four groups of university students. Participants were generally aware of the link between STIs and potential infertility and considered the discussion of this subject very relevant at their age. Knowledge about how and why STIs potentially lead to fertility complications was poor. The issues of blame relating to infertility following an STI emerged, although most participants did not think that access to free reproductive technologies after an untreated STI should be limited.

**Conclusions:**

Young people would benefit from more education in order to improve their understanding of the long-term consequences of untreated STIs, such as infertility. Participants in our sample felt these were extremely relevant and important issues for them to understand alongside current education about STIs.

## Background

Fertility problems affect approximately one in seven couples in the United Kingdom (UK) [1]. In women, infertility may be due to ovulatory problems, anatomical disorders such as damaged fallopian tubes and/or endometriosis [2]. Causes of male infertility include; abnormal semen characteristics, impaired reproductive tract, erectile dysfunction and/or ejaculatory disorders [3]. Following investigations and treatment for any aetiological factors, couples may seek assisted reproductive technology [2,3]. In 2010, 27,918 couples underwent fertility treatment in the UK [4]. Unfortunately, the success rate of in vitro fertilisation (IVF) using a female’s own fresh eggs is low at approximately 25% [5], and the process of IVF is not without its own physical and emotional challenges [6].

One of the causes of damage to both female and male reproductive systems is a delayed or untreated sexually transmitted infection (STI). The most common STIs to cause female and male infertility are chlamydia and gonorrhoea. Chlamydia is the most frequently diagnosed STI in England with 186,196 new cases diagnosed in 2011, with young people (aged 15–24 years) being at most risk [7]. Being a largely symptomless disease, it can often go undiagnosed and, therefore, untreated. Persistent chlamydia infection can potentially cause serious complications for both men and women [8]. If left untreated, women are at risk of developing pelvic inflammatory disease (PID). This is a serious condition involving inflammation of the upper female genital tract and supporting structures. It causes various complications including chronic pelvic pain, increased risk of ectopic pregnancy and tubal factor infertility (TFI) [8], which is a significant cause of infertility [9]. Of the 14,551 reasons for requiring IVF treatment given in 2010, the majority of these (other than unexplained) were for tubal factors [4]. In women, the link between infertility and past infection with chlamydia has previously been well-documented [10-14] although a more recent systematic review containing one RCT has weakened this evidence [15]. Screening programmes within England (e.g. The National Chlamydia Screening Programme) currently advise young women that chlamydia may cause infertility. The extent to which chlamydia infection impacts on male fertility is still uncertain. Chlamydia can cause inflammation of the epididymis, testes and accessory glands which can ultimately damage sperm [16]. In men, fertility problems are usually the result of reduced semen characteristics (e.g. low numbers or poor quality of sperm). Although less common than chlamydia in England, there was a 25% increase in gonorrhoea cases from 16,835 in 2010 to 20,965 in 2011 [7]. Gonorrhoea is similar to chlamydia in its transmission, diagnosis and complications [17,18].

Understanding young people’s knowledge and beliefs about the possible long-term consequences of delayed or untreated STIs is important to guide effective sexual health education. Previous research has indicated that 70% of young people feel protecting their fertility is important to them with females reporting greater concern than males [19]. With regard to young people’s knowledge and beliefs about reproductive technologies for infertility or sub-fertility, research is considerably lacking.

### Aims and objectives

Untreated chlamydia and gonorrhoea can have serious consequences in later life, particularly with regard to fertility. There is currently only limited available research regarding the key at risk age groups’ (16–24 year olds) knowledge and beliefs about the link between untreated STIs and infertility, and whether awareness of future fertility issues is of relevance amongst this age group. This study poses the following research questions: What do young people understand, and what are their beliefs, about the link between untreated STIs in particular chlamydia and gonorrhoea, and infertility in later life? Do young people consider discussions about the possible long term consequences of STIs in terms of future fertility relevant at their age?

## Methods

### Design

This study used a qualitative methodology in the form of focus groups. This was to enable discussion of differing beliefs and understanding through participant interaction, unlike other forms of qualitative research such as interviewer administered questionnaires and semi-structured interviews [20].

### Participants

Participants were young people aged between 16 and 24 years inclusive, who have been shown to be at high risk of contracting STIs [21].

### Sampling and recruitment

Groups were sampled based on two broad age groups: Further Education (Sixth Form College) students (aged approximately 16–18 years) and Higher Education (University) students (aged approximately 19–24 years). Participants were quota sampled on the basis of gender and age to obtain equal numbers of males and females and to ensure a range of ages were represented. It was anticipated that ten single sex groups would be generated. People under 16 and over 24 years old were excluded. The study aimed to recruit between six and eight participants to each group to encourage participant interaction and discussion without the group size becoming unmanageable.

### Settings

Seven further education colleges in the South East of England, UK were asked to participate. Three colleges agreed to participate (a list of colleges can be found in the acknowledgements).

Members of three universities in the same region were invited and agreed to participate. Participants were recruited in a number of different ways including emails, online adverts, posters, personal contacts and handing out flyers. Students of medicine were excluded from the study as their previous medical teaching may have led to knowledge biases in the sample.

### Procedure

Data were collected between February and March 2012. The focus groups for the college students were conducted in quiet rooms provided by the respective colleges. The university groups were held in off-campus student accommodation.

Focus groups lasted approximately 45 minutes each and two fourth year undergraduate medical students (ALG and EF) facilitated. Supervision and training by CL provided the facilitators with expertise and training in focus group methodology and practice. The key facilitator directed the majority of the discussion and encouraged interaction and exploration of all issues. The co-facilitator ensured time was kept, all participants contributed and all areas of interest were covered. The co-facilitator organised the recording equipment and went through the written consent procedures. Participants were informed that there were no right or wrong answers and that their opinion was being asked for. Discussions were based on a predetermined topic guide of broad questions with set prompts to help encourage conversation if required. In order to encourage individuals to engage in the discussion, each focus group discussion began with open questions, for example; “What does the term sexually transmitted infection mean to you?” The topic guide addressed knowledge and understanding of chlamydia and gonorrhoea (not presented here) as well as awareness of infertility and beliefs surrounding reproductive technologies.

Rules of confidentiality were agreed to ensure that details or members of the focus groups were not discussed outside of the setting. Students were given a participant information sheet (PIS) to read, a consent form to sign and an anonymous basic demographic sheet to The PIS was discussed and time was allowed for questions ensuring all members understood the information before asking them to sign for informed consent. There were no anticipated risks to participants, however, participants could leave or have a short break if they became embarrassed or upset during the discussion. Participants could be directed to the appropriate student support services for extra support if required. The focus groups were digitally recorded on two Dictaphones. Participants were invited to enter a prize draw to win a £40 Amazon voucher and complementary snacks and drinks were provided. Sexual health promotional leaflets targeted at young people were handed out at the end with local information about where to go for STI testing. Ethical approval and research sponsorships were granted from Brighton and Sussex Medical School Research Governance and Ethics Committee (11/119/LLE) and the University of Southampton (RGO Ref:8475).

### Analysis

The audio-taped discussions were transcribed verbatim by the key facilitator and reviewed for accuracy. The results were analysed using a Framework Analysis Approach [22]. Framework analysis involves the identification, classification and organisation of data according to key themes and subthemes. It is a matrix-based approach to qualitative data analysis, which uses verbatim transcripts. The transcripts were analysed by 2 coders independently, line by line by hand, and master and sub-themes were generated. Key themes that arose from the data were compared between focus groups as this allowed for comparisons and contrasts to be made across age and gender. Validity/credibility of the focus group findings was ensured by discussion between the two coders about interpretation of the data and the classification of supporting quotes into themes. A third researcher was available in case of disparities, but none were noted.

## Results

### Basic demographics

Ten single-sex focus groups (n = 60) were conducted: six with college students and four with university students (Table 1). The study included 29 males and 31 females in total (Table 2).

**Table 1:** Profile of focus group participants

**Focus group**^**1**^	**Venue**	**n**	**Gender**	**Age range (mean) in years**
CM1	College	7	Male	16–18 (17)
CF2	College	8	Female	17–18 (17)
CM3	College	5	Male	16–17 (16)
CF4	College	8	Female	17–19 (18)
CF5	College	5	Female	16–17 (16)
CM6	College	6	Male	17–18 (17)
UM7	University	6	Male	19–20 (20)
UF8	University	6	Female	18–20 (19)
UM9	University	5	Male	19–20 (19)
UF10	University	4	Female	20 (20)

^1^*C* college, *U* university, *M* male, *F* female.

**Table 2:** Characteristics of focus group participants (n = 60)

**Characteristics**	**Participants (*n*** **= 60)**^**1**^
Age range (mean)	16–20 (17.98)
Median age	18
*Gender*	
Female	31 (52%)
*Ethnicity*	
White	56 (93%)
Asian/Asian British	3 (5%)
Other ethnic group (Middle Eastern)	1 (2%)
*Highest Educational Qualification*	
<5 A*- C levels at GCSE^2^	9 (15%)
≥5 A*- C levels at GCSE	28 (47%)
A levels^3^	21 (35%)
Non-European Union	1 (2%)

^1^One male’s basic demographics missing.

^2^GCSE (General Certificate of Secondary Education)- an academic qualification taken in a number of subjects by students aged 14–16 in secondary education in England, Wales and Northern Ireland.

^3^A level (Advanced Level) - an academic qualification taken by students aged 16–18 in further education.

### Themes

We report the master and subordinate themes surrounding young people’s knowledge and beliefs about the link between untreated STIs and infertility that emerged from discussions (Table 3).

**Table 3:** A summary of the themes discussed in the focus groups

**Master theme**	**Subthemes**
Knowledge and beliefs about untreated STIs potentially causing infertility	• Awareness
	• Relevance to participants
	• Access to reproductive technologies after an untreated STI
	• Blame

### Knowledge and beliefs about untreated STIs and potential infertility

#### Awareness

In one male college group no-one was aware that untreated STIs could lead to infertility. In two of the female college groups some of the participants were aware and most, but not all, participants from one undergraduate female group knew of these possible consequences. In the remaining groups all the participants were aware to some extent although knowledge was limited. All participants that were aware of this link knew that STIs could cause infertility in women, although some participants did not know it could affect men. Whilst many participants were aware of the link between chlamydia and infertility, some did not know about gonorrhoea and potential infertility. Although some females in college groups were aware that STIs could cause infertility by blocking the fallopian tubes, most individuals did not have any understanding regarding the pathophysiological mechanism.

“Would their eggs die in the ovary?”(CM1) “Make your womb uninhabitable.”(CM1) “Does it [chlamydia] affect your hormone balance?”(CM1).

Only one participant knew that chlamydia could cause pelvic inflammatory disease (PID). None of the other participants had heard of PID. In keeping with this finding, none of the participants were aware of any of the other complications of chlamydia and gonorrhoea including ectopic pregnancies and chronic pelvic pain. Participants were also not aware of the complications for males such as epididymitis. When asked about complications, many suggested impotence, incontinence, ovarian cysts, miscarriages and endometriosis.

#### Relevance to participants

The majority of participants felt that discussions about infertility were relevant to them at their current age:

“This is the age where you have to start thinking, if I’m going to be infertile by the time I’m 30 then I need to plan my life around having children early, so I think it is really relevant.”(UF8) “Yeah like a couple of years ago I’d have said no.”(UF8).

Facilitator: “Do you worry about whether you can have children?” Participant: “Yeah.” “Yeah all the time.”(CF5) Facilitator: “Yeah, in the future or now?” Participant: “Both.”(CF5).

Many felt infertility and chlamydia should be talked about and that discussions should start at an earlier age. *“I guess so ‘cause I don’t really know a lot about what you’re talking about. I think people brush it off ‘cause it won’t happen to them so yeah it’s a big thing so this age is good.”(CF2).*

“Definitely relevant now but it should have been started earlier.”(UF10).

Some of the female participants considered that discussions regarding fertility would be of more concern to females than males.

“They’re like ‘I’m a man, I’m not going to get infertile bla bla bla oh wait I’m 30 and I’m infertile’, they don’t think about it I doubt whereas I guess it’s more in our nature, we’re supposed to reproduce.”(UF8) and “Probably now guys wouldn’t even think about it probably at this age or at an age when they’re not wanting kids but when they get to an age where they want kids I think it will be equal.”(UF10).

Although male groups generally felt that infertility was of equal issue for both sexes, some felt that females would be more concerned. *“Yeah because they’re the ones carrying the kid for nine months.”(CM3) and “I always thought that girls wanted it [children] more.”(UM7) “It’s a motherly thing they are so broody.”(UM7).*

Participants felt that careful explanations of the underlying processes would help young people understand and retain information, for example, explanations of how STIs could potentially cause infertility would aid their understanding rather than just being told the fact that they can.

“It’s more knowledge, understanding of how it works and why it happens then you like understand it.”(UM9) and “I think like you have to really make sure people understand what’s going on.”(CM6).

#### Access to reproductive technologies after an STI

In vitro fertilisation (IVF) and adoption were mentioned by most participants as means of coping with infertility. All the participants were aware of “test tube babies” and were either aware of the term IVF or knew about the processes it involved.

“Aren’t the eggs removed, fertilised and when an embryo starts to develop they are put back into the womb.”(CF4).

### Blame

There were very mixed views within and between groups regarding access to free UK National Health Service (NHS) IVF treatment if a previous STI could have been the cause of infertility. There were no consistent trends across the gender or age groups. Some participants placed blame on individuals that could have become infertile due to STIs.

“But I think if you’ve got to the stage where if you’ve had chlamydia several times then really are you in a position where you deserve to get IVF on the NHS?”(UM9) “Yeah because that shows negligence.”(UM9) and “I thought they [an infertile person following an STI] should pay up for it [IVF].”(CM3) “Suffer the consequences.”(CM3) and “That would be partly her own fault..[infertility following an STI].”(UF8).

Others felt that it would not be possible to establish fault and therefore they should be eligible for NHS treatment and some felt that the cause of infertility should not be taken into account at all.

“Yeah I think it’s equal, you can’t really judge someone on their mistakes.”(CM6).

## Discussion

The young people who took part in this study were generally aware that STIs could lead to complications with fertility, however, knowledge about the process of how STIs cause infertility was poor. As expected, older participants from the university groups generally had a better understanding than the college groups. There was a definite need, and indeed wish amongst the young people in our study, to further understand how and why STIs could potentially cause fertility complications. Participants felt these were extremely relevant and important issues for them to understand alongside current education about STIs.

Previous research has demonstrated that young people have a poor awareness of the link between STIs and infertility [19,23,24] whereas others have reported the opposite [25-27]. This disparity may primarily be due to the differences in age and educational levels of the samples, and the different methods of data collection. The present study found that whilst most people knew STIs could cause infertility in females, awareness of the effect on male fertility was lower, which is in concordance with the literature [26,27]. We found no clear gender differences in level of understanding. These data suggest that emphasis could be placed on educating young people about the effect of STIs on male as well as female fertility.

Whilst many participants were aware of the link between untreated chlamydia and possible infertility, awareness about gonorrhoea and infertility was much lower. This probably reflected participants’ poorer knowledge about gonorrhoea compared to chlamydia and has been highlighted elsewhere [28]. The National Chlamydia Screening Programme (NCSP), set up in England and Wales, UK in 2003, is a control and prevention programme for sexually active individuals under the age of 25. This programme perhaps explains the greater awareness of chlamydia than gonorrhoea amongst young people in our sample and it has been suggested that testing for gonorrhoea is added to the programme [29].

Although some females demonstrated awareness that STIs can cause infertility by blocking the fallopian tubes, most individuals did not have any understanding regarding how these STIs could result in infertility. This is in agreement with earlier research using a sample from a health centre, in which an adolescent stated, “*there is something blocking it [tube]*” but was unsure of the inflammation and scarring that causes tubal factor infertility [30]. Participants in our study felt that more education about the pathophysiology would help their understanding and perhaps lead to decreased risk taking behaviour or increased testing rates. Previous research has also found that focusing on how the bacteria can spread through the pelvic organs in the weeks following STI transmission may be more appropriate than just discussing future chances of infertility [27].

The present study found that the majority of young people felt the topic of infertility was relevant to them and that they were concerned about their future fertility. Similar results have previously been found in a study of high school students’ which reported that 70% of young people felt that protecting their fertility was important to them [19]. Our study adds to this survey data by providing in depth insight into how important and in what ways fertility is important to young people. Another study specifically about chlamydia highlighted that whilst some participants expressed a desire to protect their future fertility, “*Yes I’m worried about it. Not being able to have kids, which is something that I really want to do one day*.”, other participants showed less concern, “*If say like I was having a think one day it might pop in to my head, but I wouldn’t be in a room with someone and think ‘well I might not be able to have kid one day if I don’t (use a condom), you know*” [27]. In the present study people from university groups felt in retrospect they would not have been concerned about fertility a couple of years ago despite younger college groups stating they were currently concerned. Increasing awareness of potential infertility from untreated or delayed treatment of STIs may encourage some young people to undergo an STI check or have more regular STI screens.

There are a number of strengths and limitations to the present study. The large number of participants enabled a great quantity of detailed data to be obtained so that theoretical saturation was achieved. Many previous studies have only used university students, whereas the present study also recruited a younger and less educated cohort from a number of different institutions. Equal numbers of males and females in the present study allowed for gender comparisons to be made. For this exploratory area of research, the in-depth methodology used was more appropriate than quantitative methods. However, there are a number of limitations associated with focus group research. For example, the individuals may have been influenced by others in the group by feeling pressured into agreeing with the dominating views. The facilitators aimed to encourage less dominant participants in order to minimise this. Focus group discussions may steer towards more normative dialogues [31] than, for example, individual interviews. We do not know exactly how many of the participants knew each other, but it is highly likely that people volunteered in groups. The participants from the colleges will have known each other to varying extents. This may have influenced the nature of the interactions, although it is not uncommon for focus group respondents to know each other, due to the nature of recruitment. Participant information sheets were given out prior to the focus groups which may have provided the participants with knowledge they did not originally have. Finally, it is important to remember that the findings of this study cannot be generalised to a wider population of young people. Our samples only contained individuals in further and higher education from one geographical area of the United Kingdom. This may have biased the study towards those with more informed views than those who had left school at a younger age. With such a small proportion of ethnic groups in our sample (representative of the ethnic diversity of the geographic region which is predominantly white and of UK origin), it was not possible to tease out any ethnic or racial differences in views. Only one respondent was not originally from the UK.

## Conclusions

The present study has shown that whilst young people do have a basic awareness of issues related to STIs and infertility, they still lack a clear and consistent understanding. Sexual health education needs to begin at an earlier age and needs to focus on describing the process of how an untreated STI may result in infertility alongside emphasising the consequences, in an age-appropriate way. Education is crucial in order to protect young people from developing potentially life-altering long-term problems with fertility.

## Competing interests

The author(s) declare that they have no competing interests.

## Authors’ contributions

CDL, ALG and ERF conceived of the study, and participated in its design, coordination and analysis, and drafted the manuscript. All authors read and approved the final manuscript.
